# Birth weight and melanoma risk: a population-based case–control study

**DOI:** 10.1038/sj.bjc.6604159

**Published:** 2007-12-18

**Authors:** I Franco-Lie, T Iversen, T E Robsahm, M Abdelnoor

**Affiliations:** 1 Department for Research and Education, Center for Clinical Research, Ullevaal University Hospital, Oslo 0407, Norway; 2 Department of Oncology, The Norwegian Radium Hospital, Montebello, Oslo 0310, Norway; 3 Cancer Registry of Norway, Montebello, Oslo 0310, Norway; 4 Section for Epidemiology and Biostatistics, Department for Research and Education, Center for Clinical Research, Ullevaal University Hospital, Oslo 0407, Norway

**Keywords:** melanoma, birth weight, population-based, case–control study

## Abstract

We investigated whether lower birth weight was associated with lower risk of melanoma later in life. This population-based case–control study included all incident cases of histologically verified invasive melanoma diagnosed until 31 December 2003 in the Norwegian population born between 1967 and 1986 (*n*=709). The control group without malignant disease was established by random sampling from the same source population as the cases (*n*=108 209). Data on birth weight, gender, mother's residence and parental age at the time of birth were collected from the Medical Birth Registry of Norway and data on cancer from the Cancer Registry of Norway. The Mantel–Haenszel test of linear trend showed no trend in risk across the birth weight categories: individuals in the highest quartile of birth weight (⩾3860 g) had an odds ratio (OR) of 1.19 (95% confidence interval, CI: 0.77–1.84) compared to individuals with birth weight <2500 g. The adjusted OR was 0.81 (95% CI: 0.52–1.26) for birth weight below 2500 g (exposed). Though not statistically significant, the results suggest that low birth weight might influence the risk of melanoma later in life.

Exposure to intermittent high-dose ultraviolet (UV) radiation is widely recognised as the major cause of cutaneous malignant melanoma (hence referred to as melanoma). In two previous studies, we observed a lower incidence of melanoma in twins as compared to singletons (Iversen *et al*, 2001; Franco-Lie *et al*, 2005) as have others (Hemminki and Li, 2002; Hemminki and Chen, 2005; Neale *et al*, 2005), but for which no biological explanation is evident.

Twins and singletons differ in mean birth weight, length at birth and average duration of gestation. In the United States, mean birth weight is 3351 grams (g) for singletons and 2367 g for twins (Alexander *et al*, 1998), and Norwegian data are similar.

It has been hypothesised that birth weight can influence the cancer risk later in life. This possible association has been studied for melanoma (Andersson *et al*, 2001; McCormack *et al*, 2005; Ahlgren *et al*, 2007) and other types of cancer (Le Marchand *et al*, 1988; Tibblin *et al*, 1995; Roman *et al*, 1997; Wanderås *et al*, 1998; Vatten *et al*, 2002; Hjalgrim *et al*, 2004; Nilsen *et al*, 2005; Michos *et al*, 2007). The results are inconclusive; however, there seems to be an association between birth weight and the risk for some cancer forms.

We investigated whether lower birth weight is related to lower risk of melanoma later in life by estimating the association between birth weight and the incidence of melanoma in the Norwegian population.

## MATERIALS AND METHODS

This population-based study includes all live-born men and women (*n*=1 142 635) in Norway between 1 January 1967 and 31 December 1986. From this 20-year cohort, a case–control study was carried out.

All incident cases of melanoma diagnosed in that population before 31 December 2003 were identified (*n*=859). Melanoma was defined according to the international classification of diseases, and only cases with histologically verified invasive melanoma of primary tumour were included in the study (*n*=711). The control group is a random sample of the population, without a malignant disease, born in the same period of time as the cases and in which the individuals were all alive at the closing date 31 December 2003 (*n*=109 727).

For all the individuals, data on birth weight, gender, mother's residence and parental age at the time of birth, maternal pregnancy factors and twin status were collected from the Medical Birth Registry of Norway (MBRN). This is a population-based registry based on compulsory notification of every birth or late abortion with a gestational age of 16 weeks or more. Infants and parents can be identified thorough the national 11-digit personal identification number (Irgens, 2000).

Data on cancer were collected by linkage to the Cancer Registry of Norway, using the unique 11-digit identification number. The cancer registry is population-based (Cancer in Norway, 2006).

Cases without histological verification of the primary tumour (*n*=87) and cases of multiple cancers (*n*=61) were excluded from the analyses. Individuals lost to follow-up (controls, *n*=3) and individuals with follow-up shorter than 1 year (due to emigration or death) were also excluded (case, *n*=1; controls, *n*=1324). This 1-year limit was based on the definition of infant mortality rate (Gowen, 2006). Furthermore, individuals with missing data on birth weight (case, *n*=1; controls, *n*=191) were also excluded. The final study population includes 709 cases of melanoma and 108 209 controls without a malignant disease.

The birth weight (g) was analysed both as a continuous variable and as a categorical variable: <2500 and ⩾2500 g. The two categories were defined by the low birth weight (LBW) limit of 2500 g (Gowen, 2006). Birth weight was also analysed after distribution into five categories. The lowest category is based on the LBW limit and the others were based on quartile limits.

Norway is geographically spread from 58 to 72° latitude north and the variation in UV radiation is large. The variable ‘place of birth’, with the categories ‘North Norway’ and ‘South Norway’, was created using the separation line located approximately at 62° latitude north.

The variables ‘mother's age’ and ‘father's age’ were polytomised into quartiles, and the 75% quartile limits were used to create two levels in each variable: younger and older parents. We have missing data on father's age (cases, *n*=32 and controls, *n*=8305).

### Statistical analysis

We have used an explanatory aetiological strategy, where the association of the exposition (birth weight) and the disease (melanoma) was the major focus (Abdelnoor and Sandven, 2006). The crude associations between the exposition birth weight and the disease melanoma and between the covariates and the outcome were estimated by the odds ratios (ORs) and the 95% confidence interval limit (95% CI) (contingency tables). To calculate the effect of the continuous variables, the Student's *t*-test was used. The gradient effect between different birth weight expositions and the incidence of melanoma was done using the Mantel–Haenszel test of linear trend. Stratified analysis (Mantel–Haenszel method) was used to pinpoint effect modification via the Breslow–Day test of heterogeneity. Quantification of confounding was done by comparing the crude estimate of OR and the adjusted Mantel–Haenszel OR. A logistic multivariable model was used to control for multiconfounding (Kleinbaum *et al*, 1982). The statistical analyses were performed using SPSS Version 14.0 for Windows (SPSS Inc., Chicago, IL, USA). For the power calculation and for the test of linear trend, Epi Info for Windows Version 3.3.2, 2005 (Centers for Disease Control and Prevention, Atlanta, GA, USA) was used.

## RESULTS

A total of 709 histologically verified incident cases of invasive melanoma were diagnosed in the study population. The group of cases had gender distribution of 68.0% women and 32.1% men. The OR of melanoma for women was 2.35 (95% CI: 2.01–2.76) in relation to men. The risk of melanoma was 30% higher (OR=1.30, 95% CI: 1.09–1.55) for individuals born in the south, as compared to individuals born in the north (Table 1). Evaluation of the difference in maternal or paternal age at birth using *t*-test showed no statistically significant difference between the case and the control groups. Similarly, no difference was found when parental age was assessed as a categorical variable (Table 1). Maternal pregnancy factors such as preeclampsia/eclampsia, hypertension during pregnancy and chronic kidney disease, occurred less frequently among the cases; however, the difference was not significant. Table 1 also shows that the risk of melanoma in twins tends to be lower than in singletons (OR=0.76, 95% CI: 0.40–1.41) though not significantly, possibly due to lack of power.

### Birth weight

The controls had a lower mean birth weight, regardless of whether the genders were combined or separated for analysis (Table 1). However, the difference was not statistically significant. Individuals with a birth weight below 2500 g tended to have a reduced risk of melanoma (OR=0.86, 95% CI: 0.57–1.31). In this study population, the mean birth weights were 2640 and 3524 g for twins and singletons, respectively.

In the Mantel–Haenszel test of linear trend, the individuals in the highest quartile of birth weight (⩾3860 g) had an OR of 1.19 (95% CI: 0.77–1.84) compared to individuals in the lowest category (<2500 g) (Table 2). There was no trend in risk across the distribution of birth weight.

Results from the stratified analysis, estimating the risk of melanoma associated with birth weight, independent of gender, showed the adjusted Mantel–Haenszel OR of 0.84 (95% CI: 0.55–1.27), which corresponds to a confounding effect of –2.3%. The confounding effect for each of the other variables in the analysis (place of birth, mother's and father's ages at time of birth) was below 5%. No effect modification was observed (data not shown).

Table 3 gives the results from the multiple logistic regression analysis, estimating the effect of multiconfounders. Birth weight below 2500 g seems to reduce the risk of melanoma later in life, also when possible confounding variables such as gender, place of birth, maternal and paternal age were included in the analysis (OR=0.81, 95% CI: 0.52–1.26). The model confirmed the results from the univariable analysis (Table 1).

A power calculation was performed for the effect of OR=0.81, considering the frequency of exposition to birth weight <2500 g in controls to be 3.7% (Table 1), with a type I error of 5% and with a power of 80%. A sample size of 5864 cases of melanoma and 891 328 controls would be needed to have a 80% chance to observe a significant 19% reduction in melanoma risk in individuals with birth weight below 2500 g.

## DISCUSSION

We questioned whether individuals with a LBW were less likely to develop cutaneous malignant melanoma compared to individuals with higher birth weight.

Results from the univariable and the multivariable analyses indicate that birth weight might be associated with occurrence of melanoma, also when other possible confounders, such as gender, place of birth and parental age were included in the model.

In 2001, Andersson *et al* (2001) observed a 2-fold increase in cancer risk in the highest quintile of birth weight compared to the lowest quintile (relative risk (RR)=2.07, 95% CI: 1.22–3.50). A study published by McCormack *et al* (2005) did not find any association between birth weight and the occurrence of melanoma (hazard ratio=1.03, 95% CI: 0.85–1.26). Ahlgren *et al* (2007) observed a positive linear trend for birth weight and incidence of melanoma (847 melanoma cases) with RR of 1.04 (95% CI: 1.00–1.31). Our data confirm the results of the last study although with nonsignificance. The study of Andersson and co-workers included only six cases of melanoma, analysed together with other cancer types under the term ‘nonhormonal cancers’, and the results are, therefore, difficult to evaluate. McCormak's study included 77 melanoma cases and the question of power is pertinent.

The observed tendency to reduced melanoma risk among twins agrees with the tendency to lower risk among those with birth weight below 2500 g, as the mean birth weight for twins was 2640 g. Maternal preeclampsia/eclampsia, hypertension during pregnancy and chronic kidney disease may influence the nutrition conditions in the womb and often lead to undernutrition of the foetus. These conditions were more frequent among controls than in the case group. These observations might support our hypothesis that LBW can reduce the risk of melanoma later in life.

In the present study, women have close to 2.5-fold risk of developing melanoma compared to men. This is possibly due to the different sun exposure habits between genders, but other factors might also be involved. Individuals born in the south of Norway had a 30% higher risk of melanoma compared to individuals born in the north. This result is due to the latitude gradients in UV exposure in Norway, which previously has been shown to be reflected in the melanoma risk (Magnus, 1973; Robsahm and Tretli, 2001; Cancer in Norway, 2006). No effect was found for parental age on risk of melanoma, which agrees with the results published by Janerich *et al* (1989).

Results from the present study indicate that LBW (<2500 g) might be associated with lower risk of melanoma later in life. Birth weight is probably not a risk factor of melanoma, but it might be a marker of other relevant factors. Investigation of other exposures correlated with birth weight should therefore be investigated in the future.

## Figures and Tables

**Table 1:** Distribution of major variables in cases of malignant melanoma of the skin and controls without malignant disease

**Variables**	**Cases (*N*=709)**	**Controls (*N*=108 209)**	**OR (95% CI)**	*P*-value*
*Birth weight (grams)*				
Mean (s.d.)	3510.4 (540.9)	3507.8 (550.2)		0.900
Females: mean (s.d.)	3475.9 (533.4)	3441.3 (531.7)		0.154
Males: mean (s.d.)	3583.6 (550.7)	3567.8 (559.8)		0.671
				
*Birth weight (grams)*				
<2500 (%)	23 (3.2)	4043 (3.7)	0.86 (0.57–1.31)	0.491
⩾2500 (%)	686 (96.8)	104 166 (96.3)	1.00	
				
*Gender*				
Females (%)	482 (68.0)	51 326 (47.4)	2.35 (2.01–2.76)	<0.0001
Males (%)	227 (32.0)	56 883 (52.6)	1.00	
				
*Place of birth*				
South Norway (%)	546 (77.0)	78 007 (72.1)	1.30 (1.09–1.55)	0.004
North Norway (%)	163 (23.0)	30 202 (27.9)	1.00	
				
*Mother's age (years)*				
Mean (s.d.)	26.0 (5.1)	26.0 (5.2)		0.831
<29 (%)	524 (73.9)	76 970 (71.1)	0.87 (0.74–1.03)	0.104
⩾29 (%)	185 (26.1)	31 239 (28.9)	1.00	
				
*Father's age (years)* a				
Mean (s.d.)	29.7 (6.2)	29.7 (6.1)		0.844
<33 (%)	492 (72.7)	72 978 (73.0)	1.02 (0.86–1.21)	0.827
⩾33 (%)	185 (27.3)	26 926 (27.0)	1.00	
				
*Maternal pregnancy factors*				
Preeclampsia/eclampsia (%)	18 (2.5)	3799 (3.5)	0.72 (0.43–1.17)	1.161
Hypertension during pregnancy (%)	8 (1.1)	1487 (1.4)	0.82 (0.38–1.70)	0.575
Chronic kidney disease (%)	2 (0.3)	962 (0.9)	0.32 (0.05–1.28)	0.085
Anaemia (%)	4 (0.6)	586 (0.5)	1.04 (0.33–2.89)	0.796
				
*Twin status*				
Twins+ (%)	10 (1.4)	2009 (1.9)	0.76 (0.40–1.41)	0.380
Singletons (%)	699 (98.6)	106 200 (98.1)	1.00	

CI=confidence interval; OR=odds ratio (crude); s.d.=standard deviation; twins+=twins, triplets, quadruplets and quintuplets.

^*^All *P*-values are two sided.

a Missing data *n*=8337 individuals.

**Table 2:** Association between different levels of birth weight and occurrence of melanoma in the study population: Mantel–Haenszel test of linear trend

**Birth weight (grams) categories**	**Cases (*N*=709)**	**Controls (*N*=108 209)**	**OR**	**95% CI**
<2500	23	4043	1.00	
2500–3189	160	22 909	1.23	(0.79–1.90)
3190–3519	176	26 664	1.16	(0.75–1.79)
3520–3859	167	27 506	1.07	(0.69–1.65)
⩾3860	183	27 087	1.19	(0.77–1.84)
				
*P* for trend=0.871				

CI=confidence interval; OR=odds ratio.

**Table 3:** Birth weight and risk for malignant melanoma: adjustment for multiconfounding effect of gender, place of birth and parental age at the time of birth, using multivariate logistic model

**Variable**	**OR**	**95% CI**	*P*-value*
*Birth weight (grams)*			
<2500	0.81	0.52–1.26	0.352
⩾2500	1.0		
			
*Gender*			
Female	2.38	2.02–2.80	<0.001
Male	1.0		
			
*Place of birth*			
South Norway	1.27	1.06–1.53	0.010
North Norway	1.0		
			
*Mother's age*			
Continuous	0.99	0.96–1.01	0.236
			
*Father's age*			
Continuous	1.01	0.99–1.03	0.382

CI=confidence interval; OR=odds ratio.

^*^All *P*-values are two sided.
